# Regulation of intraocular pressure by soluble and membrane guanylate cyclases and their role in glaucoma

**DOI:** 10.3389/fnmol.2014.00038

**Published:** 2014-05-19

**Authors:** Emmanuel S. Buys, Lincoln R. Potter, Louis R. Pasquale, Bruce R. Ksander

**Affiliations:** ^1^Department of Anesthesia, Critical Care, and Pain Medicine, Anesthesia Center for Critical Care Research, Harvard Medical School, Massachusetts General HospitalBoston, MA, USA; ^2^Department of Pharmacology, University of Minnesota Medical SchoolMinneapolis, MN, USA; ^3^Department of Ophthalmology, Glaucoma Service Mass Eye and Ear Infirmary and Channing Division of Network Medicine, Harvard Medical School, Brigham and Women's HospitalBoston, MA, USA; ^4^Department of Ophthalmology, Massachusetts Eye and Ear Infirmary, Schepens Eye Research Institute, Harvard Medical SchoolBoston, MA, USA

**Keywords:** guanylate cyclase, nitric oxide, natriuretic peptides, glaucoma, open-angle, intraocular pressure

## Abstract

Glaucoma is a progressive optic neuropathy characterized by visual field defects that ultimately lead to irreversible blindness (Alward, 2000; Anderson et al., 2006). By the year 2020, an estimated 80 million people will have glaucoma, 11 million of which will be bilaterally blind. Primary open-angle glaucoma (POAG) is the most common type of glaucoma. Elevated intraocular pressure (IOP) is currently the only risk factor amenable to treatment. How IOP is regulated and can be modulated remains a topic of active investigation. Available therapies, mostly geared toward lowering IOP, offer incomplete protection, and POAG often goes undetected until irreparable damage has been done, highlighting the need for novel therapeutic approaches, drug targets, and biomarkers (Heijl et al., 2002; Quigley, 2011). In this review, the role of soluble (nitric oxide (NO)-activated) and membrane-bound, natriuretic peptide (NP)-activated guanylate cyclases that generate the secondary signaling molecule cyclic guanosine monophosphate (cGMP) in the regulation of IOP and in the pathophysiology of POAG will be discussed.

## Primary open angle glaucoma

Primary open-angle glaucoma (POAG) is a leading cause of blindness, affecting over 2.2 million patients in the US alone, and is associated with an estimated health care cost upwards of $1.5 billion/year. Vision loss, occurring due to loss of retinal ganglion cells (RGCs) and degeneration of the optic nerve, has far-reaching effects on the patient's ability to function independently, dramatically affecting quality of life from a physical, mental and social well-being perspective. Currently, there is no definitive cure for POAG and although multiple risk factors for POAG have been identified [including intra-ocular pressure (IOP), race, age, and genetic factors], the molecular signaling involved in POAG pathogenesis remains largely unknown.

Although multiple POAG risk factors have been identified, the etiology of POAG remains to be elucidated, likely because the disease can be stratified into various subtypes defined by discrete but yet unknown biochemical pathways. Two major pathophysiologic mechanisms for POAG have been proposed. In the “mechanical theory” optic neuropathy is caused by increased IOP (see “Intraocular pressure: a risk factor for POAG” below). Alternatively, a vascular component has been hypothesized to contribute to POAG pathophysiology (see “Glaucoma and vascular dysfunction” below). However, the extent to which vascular dysfunction contributes to glaucomatous optic neuropathy remains to be elucidated and is controversial (Vajaranant and Pasquale, 2012).

## Intraocular pressure: A risk factor for POAG

POAG is often associated with elevated IOP. Measurement of IOP typically requires an anesthetized ocular surface and a cooperative patient, as corneal applanation is needed for accurate measurement of this ophthalmic vital sign. While POAG is a strongly age related disease (Sommer et al., 1991; Mukesh et al., 2002) and elevated IOP is a major risk factor for this condition, IOP does not necessarily increase with age. One longitudinal analysis found that among people aged 50–59, IOP increased after 9 years of follow-up but among subjects 60 and older, a slight decrease in IOP was noted in the same time period (Wu et al., 2006). Furthermore while African heritage is an undisputed risk factor for POAG, people of African heritage do not necessarily have higher IOP than Caucasian subjects drawn from the same population (Sommer et al., 1991). While the heritability of IOP is relative highly (56–64%) (Carbonaro et al., 2009) very few common loci for this trait have been discovered thus far (Van Koolwijk et al., 2012; Ozel et al., 2014). Other factors such as body mass index (Klein et al., 1992; Wu and Leske, 1997; Oh et al., 2005), blood pressure (Klein et al., 2005), and diabetes (Wu et al., 2006) have only modest positive associations with IOP. Data regarding how IOP might change while blood pressure varies in an individual patient are lacking. When a patient (regardless of glaucoma status) transitions between a seated to supine position there is a predictable increase in IOP of ~3 mm Hg (Lee et al., 2012, 2013). Only a few medical conditions impact the level of IOP (Arevalo et al., 1996; Garcia Filho et al., 2011) but steroid exposure is notorious for leading to elevated IOP via changes in trabecular meshwork permeability (Clark et al., 1995). It is thought that IOP fluctuates more in POAG patients than controls (Sacca et al., 1998) but the literature is conflicting regarding the role of IOP fluctuation on open-angle glaucoma disease progression (Nouri-Mahdavi et al., 2004; Bengtsson et al., 2007).

IOP is determined by the balance between the production/secretion of aqueous humor (AqH) by the ciliary processes and by the drainage of AqH via the iridocorneal angle. AqH can exit the eye through various routes: the conventional, the uveoscleral, and the uveolymphatic pathways. The conventional pathway consists of the trabecular meshwork (TM), Schlemm's canal, collecting channels, and the episcleral venous system. It is generally accepted that IOP reflects the pressure necessary to overcome the intrinsic resistance to aqueous outflow that occurs at the junction where the juxtacanalicular part of the TM meets the inner wall of Schlemm's canal (Ethier et al., 1986). In uveoscleral outflow, AqH drains through the ciliary muscle (CM) and exits through the supraciliary space and across the anterior or posterior sclera, into choroidal vessels (Fautsch and Johnson, 2006). Uveoscleral outflow, accounting for anywhere between 3% and 80% of outflow depending on the species studied (4–60% in humans) (Fautsch and Johnson, 2006), is particularly impacted by age, an important observation considering the age-dependency of POAG in both patients (Kass et al., 2002, 2010; Mukesh et al., 2002; De Voogd et al., 2005; Rudnicka et al., 2006; Leske et al., 2008) and animal models (Buys et al., 2013). Incidentally, also in animal models of secondary angle-closure glaucoma, IOP and prevalence of optic neuropathy and retinal lesions increased with age (John et al., 1998; Saleh et al., 2007). More recently, the existence of a third outflow route was postulated: lymphatic channels in the stroma of the ciliary body and interstitial spaces between CM muscle bundles may function as a backup outflow system (Yucel et al., 2009). The relevance of this uveolymphatic pathway and whether NO-cGMP signaling (see “NO-cGMP signaling in the eye” below) modulates contractility of ocular lymphatic vessels remains to be determined. Importantly, a central role for NO in lymphatic function was recently identified (Liao et al., 2011). Whether the effect of NO on lymphatic contractions is mediated by cGMP remains unknown.

In contrast to what is observed in angle closure glaucoma, POAG is not associated with apparent blockage of the anterior chamber angle. In POAG, there is variable elevation of IOP associated with impaired AqH outflow that occurs despite apparently normal anterior segment anatomy and an open iridocorneal angle (Weinreb and Khaw, 2004). IOP rises gradually over time, likely as a consequence of decreased drainage of AqH. IOP-lowering treatment significantly cuts the risk of developing glaucoma in ocular hypertensives (Kass et al., 2002, 2010).

## Glaucoma and vascular dysfunction

While IOP reduction continues to be a successful treatment to reduce the progression of POAG (Leske et al., 2003; Kass et al., 2010), the pathogenesis of POAG seemingly also depends on factors other than increased IOP. Compounds that do not lower IOP dramatically may have properties that address the underlying glaucomatous disease process and therefore could be suitable therapeutic agents (Weinreb and Kaufman, 2009; Chen et al., 2011). For example, brimonidine was superior to timolol in stabilizing visual field deterioration, despite producing a similar IOP-lowering effect (Krupin et al., 2011). Also, ocular hypertension does not necessarily lead to POAG and ocular normotension does not preclude the development of POAG (Leske et al., 2001), suggesting that other pathologies, including neurologic (similar to other chronic central nervous system diseases such as Alzheimer's diseases or Multiple Sclerosis Quigley, 2011) or vascular dysfunction [e.g., as characterized by impaired retinovascular autoregulation (Feke and Pasquale, 2008) or peripheral vascular endothelial dysfunction (Henry et al., 1999; Su et al., 2008)], may contribute to the etiology of POAG. Vascular dysfunction contributes to the development of systemic hypertension (Mendelsohn, 2005; Michael et al., 2008) and several studies have provided evidence that blood pressure affects POAG risk (Kaiser and Flammer, 1991; Tielsch et al., 1995; Hulsman et al., 2007; Leske et al., 2008; Memarzadeh et al., 2010; Cherecheanu et al., 2013). In addition, the idea that vascular dysfunction contributes to the pathogenesis of POAG is based on the hypothesis that decreased perfusion of the optic nerve leads to neurodegeneration (Harris et al., 2005; Vajaranant and Pasquale, 2012).

Both impaired blood flow and impaired vascular autoregulation have been described in glaucoma patients (Flammer et al., 2002; Feke and Pasquale, 2008; Moore et al., 2008; Feke et al., 2011). Large epidemiologic studies have found adverse associations between POAG and low ocular perfusion pressure (OPP) (Hulsman et al., 2007; Leske, 2009) or low systemic blood pressure (Bonomi et al., 2000). In addition, the Early Manifest Glaucoma Trial observed that predictors of POAG disease progression included lower systolic blood and perfusion pressure (Leske et al., 2007). Especially in normal tension glaucoma patients, systemic vascular abnormalities have been described, including impaired flow- or acetylcholine-mediated vasodilation (Gasser and Flammer, 1991; Henry et al., 1999; Su et al., 2008). Also POAG patients with early paracentral visual field loss are more likely to have systemic vascular risk factors such as hypotension and migraines (Park et al., 2011, 2012). It is important to note that blood flow regulation in the optic nerve head seems to be strongly dependent on IOP and OPP (Schmidl et al., 2011). The interaction between systemic blood pressure and IOP (defining OPP) (Tielsch et al., 1995; He et al., 2011) is particularly relevant for this review, given the role of NO-cGMP signaling (see “NO-cGMP signaling in the eye” below) in both the regulation of IOP and systemic blood pressure.

## NO-cGMP signaling in the eye

**Figure 1:**
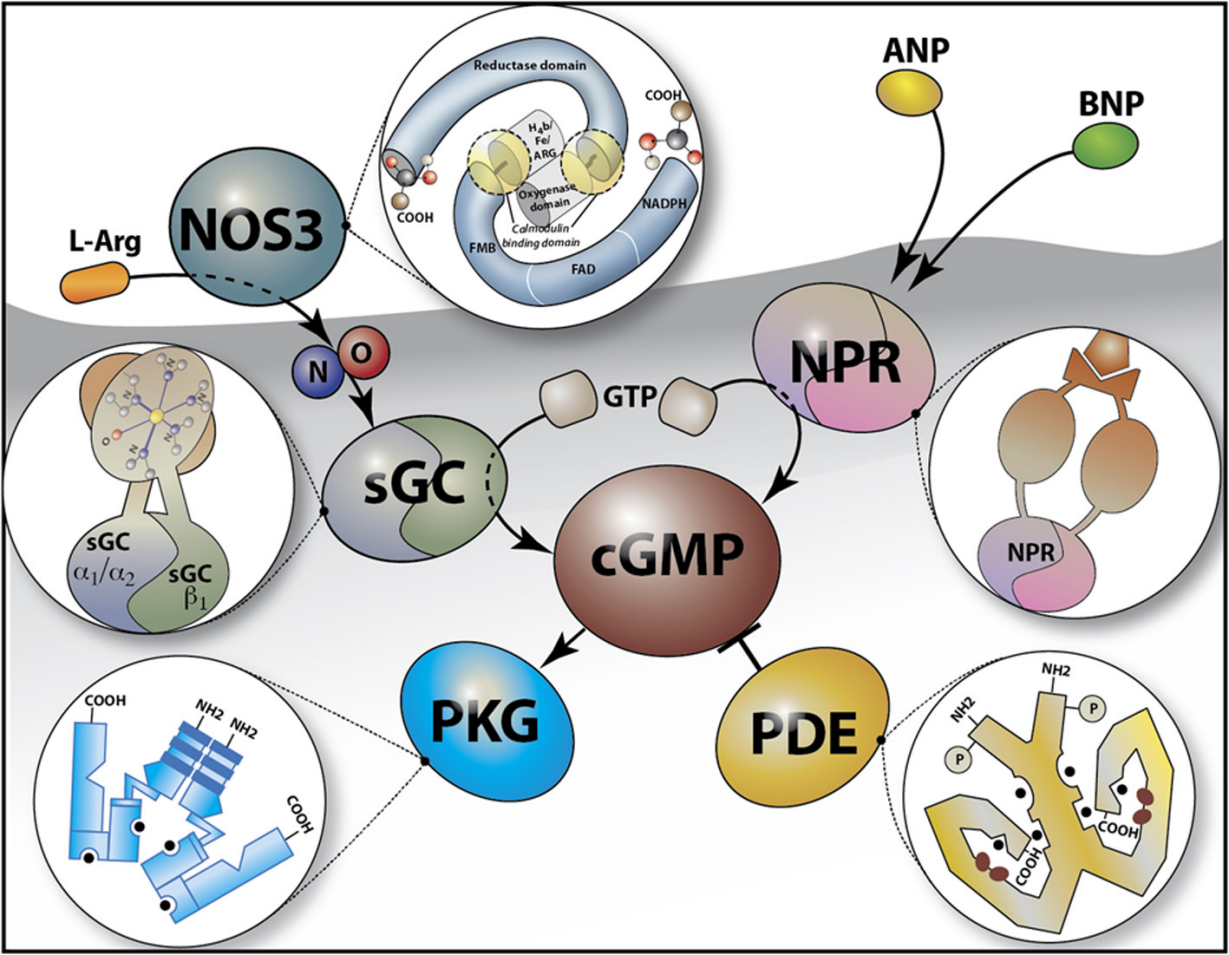
**Schematic of the Cyclic GMP (cGMP) signaling pathway**. cGMP is synthesized from GTP by soluble guanylate cyclases (sGCα_1_β_1_ or sGCα_2_β_1_) in response to nitric oxide (NO) e.g., as generated by the conversion of L-arginine (L-Arg) by NO synthase 3 (NOS3), or by the membrane guanylate cyclase (natriuretic peptide) receptors (NPR) which are activated by peptide hormones [e.g., atrial natriuretic peptide (ANP) and B-type natriuretic peptide (BNP)]. cGMP binds to and activates cGMP-dependent protein kinase G (PKG) and is hydrolyzed by phosphodiesterases (such as PDE5, the target for sildenafil). The insets depict a schematic overview of the structure of NOS3, NPR, PKG, PDE, and sGC.

NO is an exceptionally well-characterized signaling molecule, with important roles in a wide variety of physiological and pathophysiological processes, including cardiovascular homeostasis, neuronal function, and inflammation. NO is synthesized from L-arginine by a family of three enzymes referred to as NO synthases (NOSs) (Figure 1) (Moncada and Higgs, 1993). NOS2, or inducible NOS, was first identified in macrophages but has since been detected in a wide variety of cells exposed to endotoxin and cytokines. Following bacterial infection, especially with gram-negative organisms, high levels of NO are produced by NOS2. NOS1 and NOS3 were initially described to be constitutively expressed in neuronal cells and endothelial cells, respectively (Moncada and Higgs, 1993). Under physiological conditions, the low levels of NO produced by the two constitutive Ca^2+^-dependent enzymes NOS1 and NOS3 have diverse functions ranging from neurotransmission and vasodilatation to inhibition of platelet adhesion and aggregation. NO, is an important modulator of smooth muscle function and all three NOS isoforms are expressed in the eye (Table 1). Multiple studies have reported on the ability of NO to modulate resistance in the AqH outflow pathway and IOP (see “NO-cGMP-mediated regulation of IOP: implications for POAG” below). Therefore, NO is an attractive candidate as a factor that could modify both mechanical and vascular events in POAG pathogenesis.

**Table 1:** **Ocular localization of nitric oxide synthase (NOS), soluble guanylate cyclase (sGC), and NP receptors (NPR)**.

**Gene**	**Species**	**Cell- or tissue type**	**References**
NOS1	Human	Ciliary non-pigmented epithelium	Nathanson and Mckee, 1995a
	Human	ONH astrocytes, lamina cribrosa	Neufeld et al., 1997
	Monkey	Amacrine cells, rod and cone photoreceptors, RGC	Haberecht et al., 1998
	Canine	RGC	Franco-Bourland et al., 1998
	Rabbit	Amacrine cells, rod and cone photoreceptors, RGC	Haberecht et al., 1998
	Rat	Ciliary process epithelium	Yamamoto et al., 1993
	Murine	Retinal amacrine cells	May and Mittag, 2004
	Murine	Retinal amacrine cells, RGC layer somata; IPL puncta	Blom et al., 2012
	Murine	Müller cells	Chen et al., 2013
NOS2	Human	Macrophages in stroma and ciliary processes	Nathanson and Mckee, 1995a
	Human	Astrocytes	Liu and Neufeld, 2001
NOS3	Human	Longitudinal CM fibers, TM, SC	Nathanson and Mckee, 1995b
	Human	Retinal vasculature	Neufeld et al., 1997
	Human	TM	Fernandez-Durango et al., 2008
sGC	Human	RGC, IPL, ONL	Buys et al., 2013
	Human	TM cells	Ellis et al., 2009
	Rabbit	Amacrine cells, bipolar cells, cone photoreceptors, RGC	Haberecht et al., 1998
	Murine	Somata in the INL, ONL, IPL, and OPL.	Blom et al., 2012
	Murine	RGC, IPL, ONL	Buys et al., 2013
	Turtle	Amacrine cells; bipolar cells, RGC layer, IPL	Blute et al., 1998
NPR	Rabbit	Ciliary processes	Mittag et al., 1987
	Rabbit/bovine/human	Corneal endothelium	Walkenbach et al., 1993
	Rabbit/rat	Retina, choroid and ciliary process	Fernandez-Durango et al., 1995
	Human	Retina	Rollin et al., 2004
	Bovine	Choroid	Schmidt et al., 2004

ONH, optic nerve head; RGC, retinal ganglion cell; IPL, inner plexiform layer; CM, ciliary muscle; TM, trabecular meshwork; SC, Schlemm's canal; ONL, outer nuclear layer; INL, inner nuclear layer; OPL, outer plexiform layer.

NO has numerous targets, reacting with a variety of intracellular and extracellular molecules typically via thiol groups or transition metal centers (Chiamvimonvat et al., 1995; Torres et al., 1995; Davis et al., 2001; Jaffrey et al., 2001; Stamler et al., 2001). A major target of NO is the obligate heterodimer soluble guanylate cyclase (sGC) (Mergia et al., 2003, 2006; Nimmegeers et al., 2007; Vermeersch et al., 2007; Buys et al., 2008), a heme-containing heterodimeric enzyme, consisting of one α and one β subunit (Figure 1). cGMP interacts with a variety of effector proteins including cGMP-dependent protein kinases (PKGs), cGMP-regulated phosphodiesterases (PDE's), and ion channels. cGMP is also synthesized by receptor guanylate cyclases that are activated by NPs (NPRs, Figure 1). However, cGMP produced by NPRs and sGC may have differential effects, possibly due to differential spatiotemporal distributions of cGMP produced by the two guanylate cyclase families (Su et al., 2005; Castro et al., 2006; Piggott et al., 2006).

**Figure 2:**
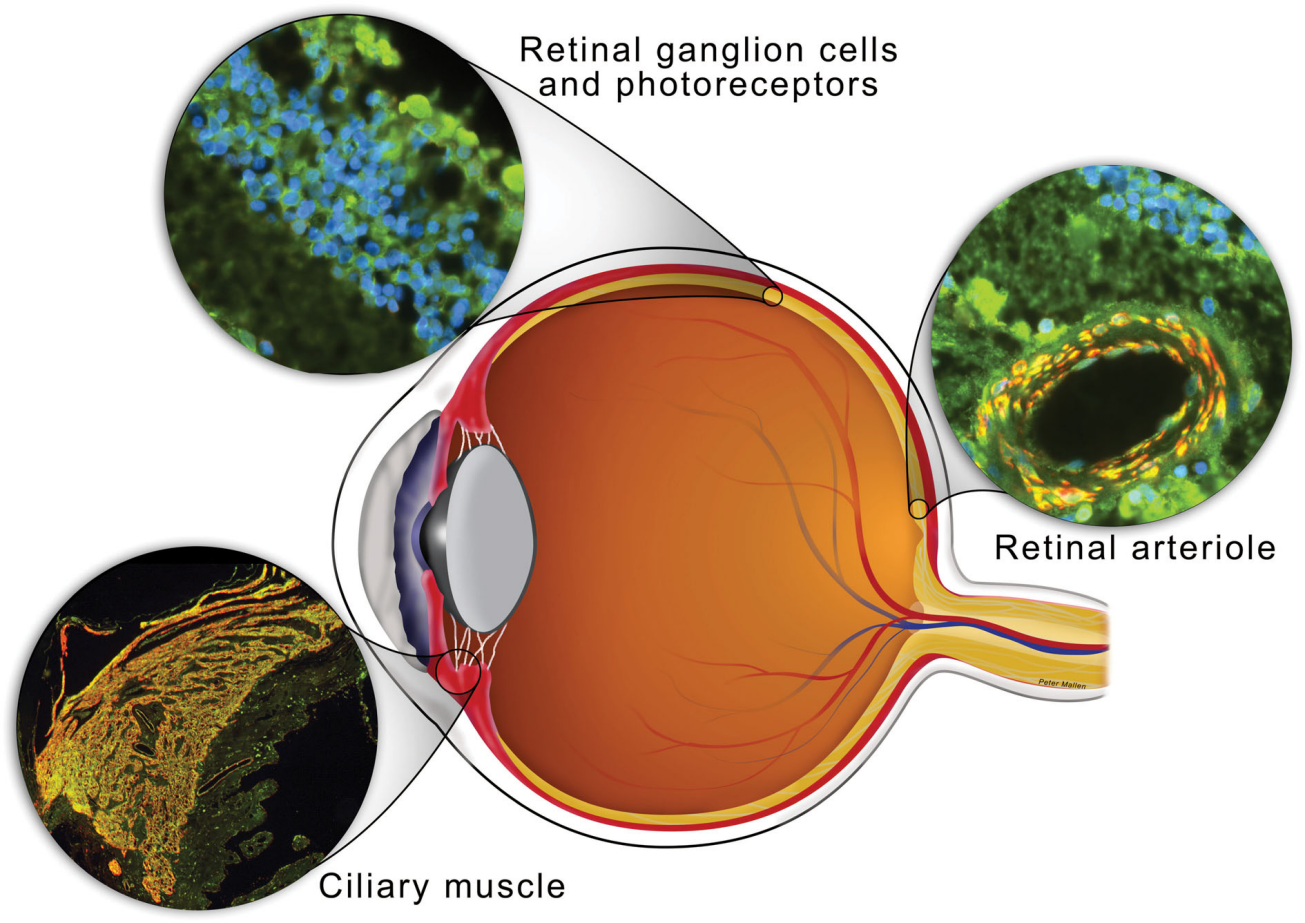
**Schematic of the eye with anatomical sites relevant for the development of POAG in which sGC is expressed indicated**. Sections through retinal ganglion cells, ciliary muscle and a retinal arteriole from a human eye were stained for sGCα_1_ (green), α-smooth muscle actin (red), and/or DAPI nucleic acid stain (blue). Both sGCα_1_ and sGCβ_1_ (not shown) co-localized with a-smooth muscle actin in ciliary muscle and the smooth muscle cell layer of a retinal arteriole (yellow in merged images). In addition, sGCα_1_ and sGCβ_1_ (not shown) expression was detected histologically in the outer nuclear layer, inner nuclear layer, and ganglion cell layer of the retina.

Two functional isoforms of sGC exist: sGCα_1_β_1_, the predominant isoform in most tissues (Mergia et al., 2003), and sGCα_2_β_1_ (Hobbs, 1997; Russwurm et al., 1998; Bamberger et al., 2001). sGCα and β subunits were shown to be expressed in several anatomical sites relevant for glaucoma (Table 1 and Figure 2). sGC activity was detected in rabbit (Haberecht et al., 1998), rat (Kajimura et al., 2003), turtle (Blute et al., 1998), and mouse retina (Blom et al., 2012; Buys et al., 2013). sGC is expressed both in retinal ganglion cells and photoreceptors and in the vascular smooth muscle layer of retinal arterioles. Finally, and most relevant to the potential ability of NO-sGC to modulate AqH outflow, sGC is abundantly expressed in isolated human TM cells (Ellis et al., 2009) and in both human and mouse CM (Buys et al., 2013). Also NP-activated membrane bound guanylate cyclase are expressed in various ocular tissues (see “NP receptors and their effects on IOP” below and Table 1).

## NO-cGMP-mediated regulation of IOP: Implications for POAG

Multiple studies have yielded evidence suggesting that NO-cGMP signaling regulates AqH outflow and IOP. For example, both an NO donor and a cGMP-analog decreased IOP (Kotikoski et al., 2002) and increased outflow facility in rabbits (Kotikoski et al., 2003). The NO-induced increase in outflow facility was impaired by treating perfused porcine eyes with the sGC inhibitor 1H-[1,2,4]oxadiazolo[4,3-a]quinoxalin-1-1 (ODQ), suggesting that the effects of NO on outflow are cGMP-dependent (Ellis et al., 2009). The non-isoform specific NOS-inhibitor L-NG-Nitroarginine methyl ester (L-NAME) decreased and an NO-donor compound increased AqH flow rate in perfused human donor eyes. This increase in flow was associated with an increase in cGMP levels measured in the perfusate, again suggesting a central role for sGC and cGMP in the ability of NO to modulate outflow (Schneemann et al., 2002). Accordingly, NO-donor compounds lower IOP (Nathanson, 1988, 1992; Schuman et al., 1994; Behar-Cohen et al., 1996; Krauss et al., 2011) and enhance tissue oxygenation of the optic nerve head in preclinical animal models (Khoobehi et al., 2011). Overexpressing NOS3 in mice lowers IOP by increasing pressure-dependent drainage (Stamer et al., 2011), confirming previous reports suggesting that the ability of NO to lower IOP is mediated by a decrease in the AqH resistance rather than a change in the rate of AqH secretion (Nathanson and Mckee, 1995b). Other possible mechanisms by which NO and cGMP can modulate outflow include regulation of Schlemm's canal cell volume (Dismuke et al., 2008; Ellis et al., 2010) and TM cell volume (Dismuke et al., 2009).

Impaired NO-cGMP signaling has been implicated in POAG. For example, NO metabolites and cGMP levels were lower in both plasma and AqH samples from POAG patients than in those from individuals without POAG (Galassi et al., 2000, 2004; Doganay et al., 2002). Also, NADPH-diaphorase (NADPH-d) reactivity, a marker for NO production, was decreased in TM, SC, and anterior longitudinal CM fibers isolated from POAG eyes (Nathanson and Mckee, 1995a) and serum levels of L-arginine analogs (endogenous NOS inhibitors) were found to be elevated in patients with advanced glaucoma (Javadiyan et al., 2012). In addition, NOS3 gene variants were associated with POAG in women (see “Genetics of POAG” below Kang et al., 2010; Magalhaes Da Silva et al., 2012). Together, these studies suggest that impaired NO-cGMP signaling can contribute to the etiology of POAG, identifying the NO-cGMP signaling pathway as a potential therapeutic target for POAG. However, the molecular mechanisms mediating cGMP's effects in the eye, and exactly how NO-cGMP signaling regulates IOP or impacts optic neuropathy remain unclear.

It is important to recognize that the role of NO-cGMP signaling in the eye is likely complex with physiological and pathophysiological effects. For example, contrary to the observations described above that NO lowers IOP, topical application of the NOS-inhibitor L-NAME reduced IOP in a rabbit model of ocular hypertension (Giuffrida et al., 2003). This reduction of IOP, observed only in hypertensive eyes and not normotensive controls, was suggested to be associated with a decreased formation of AqH production (Giuffrida et al., 2003). Opposing effects of cGMP itself on AqH dynamics have been reported: AqH flow rate decreased after intravitreal administration of the cell permeable cGMP analog 8-Br-cGMP but increased after intra-cameral administration of 8-Br-cGMP, underscoring that the route of administration of any drug aimed at lowering IOP or preventing POAG progression may significantly determines its therapeutic efficacy (Kee et al., 1994).

## No modulates RGC viability

NO can also exert a direct neurotoxic effect on RGC's (Morgan et al., 1999; Takahata et al., 2003). For example, in acute angle-closure glaucoma patients, higher levels of NO in AqH were postulated to contribute to RGC and optic nerve damage (Chang et al., 2000; Chiou et al., 2001). Similarly, in a rat model of chronic glaucoma, elevated retinal NOS1 expression was suggested to contribute to cytotoxicity and selective RGC loss (Park et al., 2007). Furthermore, NO generated by NOS2 can contribute to RGC death seen in response to increased IOP: treatment of rats with chronically elevated IOP with an inhibitor of NOS2 prevented RGC loss (Neufeld et al., 1999, 2002). More clinically relevant, expression of NOS2 was increased in the TM of POAG patients and activity of NOS2 in the TM of patients with POAG was reported to be proportional to the observed visual field defects (Fernandez-Durango et al., 2008). Together, these data could be interpreted to suggest that inhibiting NOS activity may be useful for the treatment of glaucoma by protecting RGCs from stress (e.g., as associated with elevated IOP). However, as described in the studies referred to above, there is ample evidence that impaired NO-cGMP signaling contributes to the pathogenesis of POAG. Together with the fact that NO has multiple downstream targets, of which sGC is arguably a predominant one, these observations support focusing on sGC and cGMP as therapeutic targets in POAG (see “The NO-cGMP pathway as a therapeutic target for POAG” below).

## Modulation of TM and CM contractility by NO-cGMP and its relevance in IOP regulation

It is generally accepted that the TM and CM are active structures rather than passive filters. The CM is a smooth-muscle like structure and the TM contains contractile smooth-muscle-specific alpha-actin filaments. Both are involved in fine-tuning the regulation of AqH outflow (Erickson-Lamy et al., 1991; Wiederholt, 1998; Wiederholt et al., 2000). Several studies (reviewed in Wiederholt et al., 2000) have suggested the existence of a functional antagonism between the TM and the CM, and TM resistance may be regulated by contractile state of CM (Nathanson and Mckee, 1995b): contraction of the CM would decrease TM resistance, increase AqH outflow and decrease IOP.

Alterations in smooth muscle contractility have previously been implicated in glaucoma. For example, RhoA-Rho kinase-mediated signaling, which regulates the phosphorylation status of myosin light chain, thereby directly influencing TM contraction, was reported to influence AqH drainage (Russ et al., 2010). Inhibiting Rho kinase, an enzyme that plays a critical role in regulating the contractile tone of smooth muscle tissues, increased AqH outflow in various animal models (Honjo et al., 2001; Rao and Epstein, 2007; Lu et al., 2008). The ability of Rho-kinase inhibitors to lower IOP is currently being tested in clinical trials (Chen et al., 2011; Tanihara et al., 2013).

The presence of NOS3 and sGC in the CM and TM (Table 1) suggest that the NO-sGC pathway may modulate outflow resistance by regulating CM and TM contractility. In fact, both NO-donor compounds and a cGMP analog were previously demonstrated to regulate the contractile state of bovine CM and TM in organ bath experiments (Wiederholt et al., 1994; Masuda et al., 1997; Kamikawatoko et al., 1998). The ability of NO-cGMP to regulate smooth muscle cell relaxation is well known. For example, vascular reactivity was attenuated in NOS3 mutant mice (Atochin et al., 2007) and in sGCα^−/−^_1_ mice (Mergia et al., 2006; Nimmegeers et al., 2007). NO-cGMP signaling (facilitating smooth muscle relaxation Surks et al., 1999) and RhoA-Rho kinase signaling (causing smooth muscle contraction Bennett et al., 1988) have opposing effects on smooth muscle function and NO-cGMP signaling controls activity of the RhoA-Rho kinase pathway (Sauzeau et al., 2003). Together, these findings raise the possibility that impaired NO-cGMP signaling, previously suggested to participate in the regulation of AqH outflow and IOP (see “NO-cGMP-mediated regulation of IOP: implications for POAG” above) has a similar impact on AqH drainage as does increased RhoA-signaling: both could result in augmented contractility of the smooth muscle-like CM, thereby potentially decreasing AqH drainage and increasing IOP (Honjo et al., 2001).

There is, however, an apparent paradox in the dynamic regulation of IOP by the CM and the role of NO-cGMP therein: impaired NO-cGMP signaling would be expected to result in a decreased ability of the SMC-like CM to relax, just as it does in other SMC-like structures (Mergia et al., 2006; Friebe et al., 2007; Nimmegeers et al., 2007; Vanneste et al., 2007; Decaluwe et al., 2010), and hence decrease TM resistance. Importantly, there are two types of CM in humans: circular, involved in accommodation, and longitudinal (Flugel et al., 1990; Sharif et al., 2004). The effect of CM contraction and relaxation on IOP is not uniform: the circular and longitudinal components of the CM appear to have opposite roles in controlling IOP. Contraction of the circular muscle via muscarinic stimulation (e.g., in response to pilocarpine) causes tightening of the trabecular ring leading to an opening of the trabecular juxtacanalicular tissue, decreased resistance, and increased outflow facility, ultimately lowering IOP (Kaufman, 2008). On the other hand, there is evidence that contraction of the longitudinal muscle can impair outflow and increase IOP. The latter was demonstrated in patients that suffered significant blunt trauma where the circular CM is disabled without affecting the longitudinal muscle. When these patients were treated with pilocarpine, the contraction of the longitudinal muscle paradoxically increased IOP (Bleiman and Schwartz, 1979). Therefore, relaxation of the posterior longitudinal muscle in the CM (e.g., with Rho-kinase inhibitors Honjo et al., 2001) likely has an outflow enhancing effect, lowering IOP. On the other hand, decreased relaxation of longitudinal CM fibers associated with impaired NO-cGMP signaling (e.g., in sGCα^−/−^_1_ mice, see “sGC-deficient mice: a model of elevated IOP and POAG” below), would result in increased IOP. In addition, repeated contraction and NO-mediated relaxation of any CM component may decreases outflow resistance and lower IOP, likely due to a direct mechanical effect on the TM. Lack of NO-sGC-cGMP signaling in the CM may prevent flushing of the TM, possibly resulting in altered (increased) outflow resistance.

## The NO-cGMP pathway as a therapeutic target for POAG

The NO-cGMP pathway can be perturbed by a variety of mechanisms. Decreases in NOS3 expression or uncoupling of NOS3 result in lower NO levels (Takimoto et al., 2005). Alternatively, NO bioavailability can be reduced by interaction of NO with reactive oxygen species (ROS) (Munzel et al., 2010). sGC itself can be converted to an NO-insensitive state as a consequence of increased oxidative stress: it is conceivable that the mechanism by which increased oxidative stress results in POAG (Majsterek et al., 2011a) may involve direct oxidation and inactivation of sGC (Stasch et al., 2006). Also, sGC expression can be repressed under pro-inflammatory conditions (Marro et al., 2008). The latter is relevant based on the hypothesis that inflammation contributes to the development of glaucoma (Vohra et al., 2013). Lastly, NO-cGMP signaling can be modulated by genetic variation (see “Genetics of POAG” below) (Kang et al., 2010, 2011; Ehret et al., 2011; Buys et al., 2013). Whether any of the mechanisms that alter activity of the NO-cGMP pathway contribute to the etiology of POAG remains a matter of active investigation. Nonetheless, the studies described here and in the “Modulation of TM and CM contractility by NO-cGMP and its relevance in IOP regulation” section certainly imply the potential usefulness of focusing on NO-cGMP signaling as a therapeutic approach to the treatment of POAG.

Various therapeutic agents have been and are being developed to enhance NO-sGC-cGMP signaling. NO-independent sGC stimulators are already being clinically tested and have been approved for treating cardiovascular disease (Ghofrani et al., 2013). These compounds could conceivably be developed to treat POAG. NO-independent and heme-independent sGC activators preferentially activate sGC when the heme is oxidized or missing (Stasch et al., 2006). The selectivity of sGC activators for oxidized/heme-free sGC could be exploited therapeutically to preferentially target diseased tissue (Boerrigter et al., 2007), especially since oxidative stress may have a pathogenic role in POAG (Majsterek et al., 2011a). Another advantage of specifically activating sGC is that it bypasses any toxicity associated with activating NOS (see “NO-cGMP-mediated regulation of IOP: implications for POAG” above). In addition, targeting sGC rather than NOS circumvents concerns related with reduced NO bioavailability (e.g., in a setting of increased oxidative stress) and allows for a more specific approach since the biological actions of NO are not only mediated by cGMP but also by cGMP-independent mechanisms.

Other potential therapeutic approaches specifically aimed at cGMP signaling that may be developed for treatment of POAG include targeting other enzymes that control GMP levels and which are abundantly present in the eye, including cGMP-catabolizing PDE's (Francis et al., 2011) and NP activated membrane bound guanylate cyclades (see “NP receptors and their effects on IOP” below, Mckie et al., 2010). Pilot studies testing the ability of the PDE5 inhibitor sildenafil to lower IOP were not successful. A single oral dose of 50–100 mg sildenafil failed to impact IOP in POAG patients (Grunwald et al., 2001) and healthy volunteers (Vobig et al., 1999; Sponsel et al., 2000), but did increase blood flow in both healthy subjects (Sponsel et al., 2000; Foresta et al., 2008) and patients with systemic vascular dysfunction (Koksal et al., 2005). Also chronic treatment with sildenafil (twice per week for 3 months) failed to impact IOP (Dundar et al., 2006). It is noteworthy that the cohorts in which the effect of sildenafil on IOP was studied were of limited size (5–15 subjects). It is conceivable that these studies were underpowered to detect small decreases in IOP associated with sildenafil treatment. This is a relevant concern since modest changes in IOP have been reported to impact POAG risk in humans. For example, a 2-mmHg difference in IOP distinguished between progression and non-progression in POAG patients (Leske et al., 2007; Konstas et al., 2012). Also, most of the cohorts studied consisted of men only. In light of a possible gender-specificity of POAG (Vajaranant et al., 2010; Pasquale and Kang, 2011; Tsai et al., 2012) and the role of NO-cGMP signaling therein (Kang et al., 2010), it would be of interest to see whether preventing cGMP catabolism by PDE5 would impact IOP in women. Finally, the half-life of sildenafil is rather limited (~4 h) (Smith et al., 2013). It may be worthwhile testing the effect on IOP of other PDE5 inhibitors with longer half-lives (e.g., tadalafil with a half-life of ~17 h Smith et al., 2013) and/or performing a carefully controlled dose response experiment. Finally, various methods to deliver the drug should be tested. Whether pharmacological modulation of the activity of enzymes that ultimately modulate cGMP levels may prevent POAG progression remains to be determined.

## sGC-deficient mice: A model of elevated IOP and POAG

Mice lacking the α_1_ subunit of the NO receptor soluble guanylate cyclase (sGCα^−/−^_1_ mice) were recently reported to represent a novel and translatable animal model of POAG, characterized by thinning of the retinal nerve fiber layer (RNFL) and loss of optic nerve axons (Buys et al., 2013). The optic neuropathy associated with sGCα_1_—deficiency was accompanied by modestly increased IOP, on average 2 mmHg higher in 39 ± 14 weeks old female sGCα^−/−^_1_ mice than in age-matched female wild-type (WT) mice. No IOP difference was detected between age-matched 15 ± 6 weeks old female sGCα^−/−^_1_ and age-matched WT mice: age was a predictor of elevated IOP in female sGCα^−/−^_1_ but not in WT mice. The increase in IOP was accompanied by a decrease in AqH turnover in the context of an open iridocorneal angle: sGCα^−/−^_1_ mice presented with a normal ciliary body, a well-defined TM, and a patent Schlemm's canal. In addition, spectral domain optical coherence tomography analysis of the iridocorneal angle did not reveal any evidence for angle-closure in sGCα^−/−^_1_ mice. Similarly, biomicroscopy did not reveal any evidence of exfoliation syndrome, pigment dispersion syndrome, or other conditions that could produce elevated IOP.

Highlighting the multi-faceted role of NO-cGMP signaling in ocular (patho)physiology, retinal vascular dysfunction was observed in sGCα^−/−^_1_ mice. Other studies had postulated that vascular dysfunction contributes to the etiology of POAG (Henry et al., 1999; Feke and Pasquale, 2008; Su et al., 2008) and systemic vascular dysfunction had been previously reported in sGCα^−/−^_1_ mice (Nimmegeers et al., 2007; Atochin et al., 2010; Buys et al., 2012). Whether retinal vascular dysfunction underlies the retinal degeneration and optic neuropathy in sGCα^−/−^_1_ mice remains to be determined. Vascular dysfunction may also contribute to the observed increases in IOP in sGCα^−/−^_1_ mice, possibly by impairing uveolymphatic outflow (see “Intraocular pressure: a risk factor for POAG” above). Finally, it cannot be excluded that loss of RGCs in sGCα^−/−^_1_ mice is caused by a direct effect of sGC-deficiency on RGCs, possibly modulating the susceptibility of RGCs to stress imposed by increased IOP or vascular dysfunction. Taken together, targeting sGC may represent a multi-pronged approach, aimed at lowering IOP, ameliorating vascular function, and protecting RGCs from stress-induced dysfunction and death. To test this hypothesis, additional studies, including in animal models of POAG, such as the sGCα^−/−^_1_ mouse model, are required.

## Genetics of POAG

First-degree relatives of glaucoma patients have a 22% risk of developing glaucoma versus a 2% lifetime risk of relatives of controls (Wolfs et al., 1998). In recent years, familial aggregation, genome wide and candidate gene association studies have uncovered an important genetic component to POAG (Table 2). Several dozen genetic loci have been linked to POAG (Fan et al., 2006). The role of many of the identified genes in the etiology of POAG, some of which were identified in single studies that need to be replicated, remains controversial. Among the genes identified to date, three are involved in the nitric oxide (NO)-cGMP system, highlighting a central role of this signaling pathway in the development of POAG. First, variants were identified in or near the genes encoding caveolin 1 and 2 (*CAV1/CAV2*) (Thorleifsson et al., 2010; Wiggs et al., 2011). Caveolins modulate the ability of NOS3 to generate NO (Mineo and Shaul, 2012). At least one variant in the *CAV1/CAV2* locus was subsequently found to be associated with increased IOP (Van Koolwijk et al., 2012; Ozel et al., 2014). Second, A candidate gene association study in 527 incident cases and 1543 controls revealed interactions between *NOS3* gene variants, potentially affecting expression and/or activity of NOS3, and high tension POAG in females (Kang et al., 2010). In addition, a functional NOS3 polymorphism (T-786C) was associated with POAG and appears to interact with gender and age in modulating the risk of POAG (Magalhaes Da Silva et al., 2012). The same variant also affects the interaction of systemic hypertension and cigarette smoking with POAG risk, highlighting the complex gene-environment interactions that impact the etiology of POAG (Kang et al., 2011). And thirdly, in a recent candidate gene association study in POAG patients from the GLAUGEN cohort (Wiggs et al., 2011), a variant (rs11722059) was identified in the *GUCY1A3/GUCY1B3* locus (containing the genes encoding the α_1_ and β_1_ subunits of sGC, arranged in tandem) (Wiggs et al., 2011). Intriguingly, the association was only present in POAG characterized by early paracentral visual field loss (Buys et al., 2013). Early paracentral visual field loss is a subtype of POAG previously postulated to be associated with ocular vascular dysregulation (Park et al., 2011). This was a particularly interesting finding in light of the identification of sGCα^−/−^_1_ mice with systemic and retinal vascular dysfunction, as a model of POAG with moderately elevated IOP: as described above, POAG patients with early paracentral visual field loss tend to have more frequent systemic vascular risk factors (Park et al., 2011, 2012), and low OPP is a risk factor for POAG (Leske, 2009; Cherecheanu et al., 2013). In addition, rs11722059 is in linkage disequilibrium with a *GUCY1A3/GUCY1B3* variant associated with blood pressure in a large genome wide association study (GWAS) (Ehret et al., 2011). However, the extent to which vascular dysfunction contributes to glaucomatous optic neuropathy remains to be elucidated (Flammer et al., 2002; Vajaranant and Pasquale, 2012).

**Table 2:** **POAG-associated genes**.

**Gene**	**Association**	**References**	**Remarks**
AGTR2	NTG	Hashizume et al., 2005	CGAS, interaction with gender
APOE	POAG	Copin et al., 2002	CGAS
ASB10	POAG	Pasutto et al., 2012	Family-based linkage study
ATOH7	POAG	Ramdas et al., 2011; Chen et al., 2012	CGAS, interactive effect with RFTN1
C7	POAG	Scheetz et al., 2013	GWAS
CAV1/CAV2	POAG	Thorleifsson et al., 2010	GWAS, also associated with IOP (Ozel et al., 2014)
	POAG	Wiggs et al., 2011	CGAS, interaction with gender, also associated with IOP (Ozel et al., 2014)
CDKN1A	POAG	Tsai et al., 2004	CGAS
CDKN2B-AS1	POAG	Ramdas et al., 2011	CGAS
	POAG	Burdon et al., 2011	GWAS, also associated with IOP (Ozel et al., 2014)
	POAG	Nakano et al., 2012	GWAS, also associated with IOP (Ozel et al., 2014)
	POAG, NTG, XFG	Wiggs et al., 2012	GWAS, also associated with IOP (Ozel et al., 2014)
CYP1B1	JOAG	Vincent et al., 2002	CGAS
EDNRA	NTG	Ishikawa et al., 2005	CGAS
ELOVL5	NTG	Meguro et al., 2010	GWAS
GAS7	POAG	Van Koolwijk et al., 2012	CGAS, SNP identified in IOP GWAS
GLC1B	POAG	Stoilova et al., 1996	Family-based linkage study
GSTM1	POAG	Juronen et al., 2000	CGAS
HK2	POAG and NTG	Shi et al., 2013	CGAS
IGF2	POAG	Tsai et al., 2003	CGAS
IL1B	POAG	Lin et al., 2003b	CGAS
LOXL1	XFG	Thorleifsson et al., 2007	GWAS, not associated with POAG
MMP1	POAG	Majsterek et al., 2011b	CGAS
MTHFR	POAG	Junemann et al., 2005	CGAS
MYOC	JOAG	Sheffield et al., 1993; Stone et al., 1997	Family-based linkage study
NCK2	NTG	Shi et al., 2013	CGAS
NOS3	POAG	Tunny et al., 1998	CGAS
	HTG	Kang et al., 2010	CGAS, interaction with gender and hormone use
	POAG	Kang et al., 2011	CGAS, interaction with hypertension and smoking
NPPA	POAG	Tunny et al., 1996	CGAS
OCLM	POAG	Fujiwara et al., 2003	CGAS
OPA1	NTG	Aung et al., 2002	CGAS
OPTN	NTG	Sarfarazi et al., 1998; Rezaie et al., 2002	Family-based linkage study
Six1/Six6	POAG	Ramdas et al., 2011	CGAS
	POAG	Wiggs et al., 2012	GWAS, also associated with IOP (Ozel et al., 2014)
SRBD1	NTG	Meguro et al., 2010	GWAS
TAP1	POAG	Lin et al., 2004	CGAS
TMCO1	POAG	Burdon et al., 2011	GWAS, also associated with IOP (Van Koolwijk et al., 2012; Ozel et al., 2014)
TNF	POAG	Lin et al., 2003a	CGAS
TP53	POAG	Lin et al., 2002	CGAS
WDR36	POAG	Monemi et al., 2005	CGAS

AGTR2, Angiotensin II receptor type 2; APOE, Apolipoprotein E; ASB10, ankyrin repeat and SOCS box-containing 1; ATOH7, atonal homolog 7; C7, complement component 7; CAV1, caveolin 1; CAV2, caveolin 2; CDKN1A, cyclin-dependent kinase inhibitor 1A; CDKN2B-AS1, CDKN2B antisense RNA 1; CGAS, candidate gene association study; CYP1B1, cytochrome P450 subfamily I polypeptide 1; EDNRA, endothelin receptor type A; ELOVL1, elongation of very-long-chain-fatty acids 1; GAS7, growth arrest-specific protein 7; GLC1B, glaucoma 1, open angle, B; GSTM1, glutathione S-transferase mu-1; GWAS, genome-wide association study; HK2, hexokinase 2; HTG, high-tension glaucoma; IGF2, insulin-like growth factor II; IL1B, interleukin 1-beta; IOP, intra-ocular pressure; JOAG, juvenile open angle glaucoma; LOXL1, lysyl oxidase-like 1; MMP1, matrix metalloproteinase 1; MTHFR, 5,10-methylenetetrahydrofolate reductase; MYOC, myocilin; NCK2, NCK adaptor protein 2; NOS3, nitric oxide synthase 3; NPPA, natriuretic peptide precursor; NTG, normal-tension glaucoma; OCLM, oculomedin; OPA1, optic atrophy 1; OPTN, optineurin; POAG, primary open angle glaucoma; RFTN1, raftlin lipid raft linker 1; Six1/Six6, six homeobox1/6; SRBD1, S1 RNA-Binding Domain-Containing Protein 1; TAP1, transporter ATP-binding cassette major histocompatibility complex 1; TMCO1, transmembrane and coiled-coil domains 1; TNF, tumor necrosis factor; TP53, tumor protein p53; WDR36, WD repeat domain 36; XFG, exfoliation glaucoma.

For neither the *NOS3* or *GUCY1A3/GUCY1B3* variants, an association with IOP was detected. Whether this truly means that there is no association with the identified variants remains unsure: it is conceivable that the GWAS and candidate gene association studies discussed were underpowered to detect small effect on IOP. For example, the GWAS in which the sGC blood pressure variant was identified included 200,000 subjects (Ehret et al., 2011). In contrast, the studies aimed at identifying associations with POAG and IOP (like systemic blood pressure a continuous variable) were much smaller in scope (hundreds to several thousand subjects).

## NP receptors and their effects on IOP

There are three NPs in mammals: atrial NP, B-type NP and C-type NP (Potter et al., 2009). A separate gene encodes each NP, and mouse “knock out” experiments have shown that each NP has unique functions, although all members stimulate vasorelaxation. ANP and BNP are cardiac endocrine hormones that decrease blood pressure and volume. CNP is a paracrine-signaling molecule that stimulates long bone growth, causes bifurcation of neurons in the spinal cord and inhibits meiosis in the oocyte.

There are also three known NP peptide receptors (Potter, 2011a,b,c). The natriuretic clearance receptor (NPR-C) controls the local concentration of all three natriuretic peptides through an undefined receptor-mediated internalization and degradation process, but it has also been reported to signal through a G- protein-dependent pathway. The primary signaling receptor for ANP and BNP is guanylate cyclase-A (GC-A), which is also known as natriuretic peptide receptor-A or NPR1. It consists of an extracellular ligand-binding domain, a single membrane-spanning region and intracellular guanylate cyclase domain. GC-B, also known at NPR-B or NPR2, is the primary signaling receptor for CNP and it is topologically and structurally similar to GC-A. The vast majority of NPs functions are mediated through elevation of intracellular cGMP concentrations synthesized by the guanylate cyclase domains of GC-A or GC-B.

Early studies reported that binding sites for ^125^I-ANP were found in rat and rabbit ciliary processes (Bianchi et al., 1986). Shortly thereafter, topical, intracameral and intravitreal application of ANP was shown to decrease IOP in rabbits (Sugrue and Viader, 1986). The pressure reductions were longer lasting with the intravitreal treatments but even the topical application reduced IOP. Additional studies found that ANP stimulated guanylate cyclase activity in ciliary processes of the rabbit eye and that intravitreous injection of ANP decreased IOP in the rabbit eye for up to 24 h (Mittag et al., 1987). The reduction of IOP by intravitreal ANP was later correlated to a decrease in AqH flow in the rabbit eye (Korenfeld and Becker, 1989).

Relatively low concentrations of CNP (~2 nmol/L) were also shown to increase cGMP concentrations in the AqH of the rabbit eye and reduce IOP in rabbit eyes by a process associated with an increase in total outflow facility (Takashima et al., 1998). In the same study, CNP-like immunoreactivity was detected in rabbit and porcine AqH at about two-fold higher concentrations than that found in plasma. In a more recent study, CNP was shown to be a more potent reducer of IOP than ANP in rabbit eyes, and a ring-deleted analog of ANP that blocked binding to NPR-C and increased the concentration of all endogenous natriuretic peptides, maintained IOP reductions longer that other natriuretic peptides (Fernandez-Durango et al., 1999). Thus, whether the reductions in IOP observed by early investigators were due to cross-activation of GC-B or whether activation of GC-B and GC-A leads to decreased IOP is not known. Regardless, CNP is the most potent natriuretic peptide for reducing IOP in mammals, which suggests that the degradation resistant CNP analog developed for the treatment of achondroplasia may be an ideal peptide-based molecule to reduce IOP in the clinic (Lorget et al., 2012).

## Conclusion

In conclusion, POAG remains a major cause of blindness in the USA and worldwide. The identification of new therapeutic targets for the treatment of POAG has been hampered by lack of understanding of the etiology of POAG and the limited number of animal models available that likely represent only a small subset of human POAG cases. There continues to be an urgent need for biomarkers that allow to detect/diagnose/track POAG progression and treatment efficacy. Also, the lack of a definitive cure underscores the need to develop novel therapeutic approaches for POAG.

Evidence obtained from animal models indicates that cGMP signaling plays an important role in POAG pathogenesis. These findings are supported by genetic studies of IOP and POAG risk in human subjects, highlighting the relevance of cGMP signaling in the development of POAG. Strategies aimed at modulating cGMP levels may constitute a pharmacological approach for a disease for which no definitive cure is currently available. However, additional studies are required to unequivocally determine the role of impaired cGMP signaling in POAG and to advance bench-to-bedside translation of available cGMP-enhancing reagents.

### Conflict of interest statement

The authors declare that the research was conducted in the absence of any commercial or financial relationships that could be construed as a potential conflict of interest.
